# Metabolomics differentiation of canola genotypes: toward an understanding of canola allelochemicals

**DOI:** 10.3389/fpls.2014.00765

**Published:** 2015-01-09

**Authors:** M. Asaduzzaman, James E. Pratley, Min An, David J. Luckett, Deirdre Lemerle

**Affiliations:** ^1^School of Agricultural and Wine Sciences, Faculty of Science, Charles Sturt UniversityWagga Wagga, NSW, Australia; ^2^Graham Centre for Agricultural Innovation, Charles Sturt UniversityWagga Wagga, NSW, Australia; ^3^Faculty of Science, Charles Sturt UniversityWagga Wagga, NSW, Australia; ^4^New South Wales Department of Primary IndustriesWagga Wagga, NSW, Australia

**Keywords:** *Brassica napus*, rapeseed, weed, root exudates, LC-QTOF-MS and metabolomics

## Abstract

Allelopathy is one crop attribute that could be incorporated in an integrated weed management system as a supplement to synthetic herbicides. However, the underlying principles of crop allelopathy and secondary metabolite production are still poorly understood including in canola. In this study, an allelopathic bioassay and a metabolomic analysis were conducted to compare three non-allelopathic and three allelopathic canola genotypes. Results from the laboratory bioassay showed that there were significant differences among canola genotypes in their ability to inhibit root and shoot growth of the receiver annual ryegrass; impacts ranged from 14% (cv. Atr-409) to 76% (cv. Pak85388-502) and 0% (cv. Atr-409) to 45% (cv. Pak85388-502) inhibition respectively. The root length of canola also differed significantly between genotypes, there being a non-significant negative interaction (*r* = -0.71; *y* = 0.303x + 21.33) between the root length of donor canola and of receiver annual ryegrass. Variation in chemical composition was detected between organs (root extracts, shoot extracts) and root exudates and also between canola genotypes. Root extracts contained more secondary metabolites than shoot extracts while fewer compounds were recorded in the root exudates. Individual compound assessments identified a total of 14 secondary metabolites which were identified from the six tested genotypes. However, only Pak85388-502 and Av-opal exuded sinapyl alcohol, *p*-hydroxybenzoic acid and 3,5,6,7,8-pentahydroxy flavones in agar growth medium, suggesting that the synergistic effect of these compounds playing a role for canola allelopathy against annual ryegrass *in vitro*.

## INTRODUCTION

Weed control options for canola in Australia have been improved considerably with the development of a wide range of herbicide–tolerant cultivars with resistance to triazine, imidazolinone or glyphosate herbicides. The implementation of glyphosate-tolerant canola has changed the pattern of herbicide use, decreasing the use of other herbicides, and has given growers an efficient and simple solution for weed control worldwide (Harker et al., 2000; Beckie et al., 2011). Unfortunately, the use of herbicides in herbicide-tolerant canola cultivars has encouraged weeds to evolve herbicide-resistance (Powles et al., 1998; Heap, 2002). The ubiquitious weed annual ryegrass (*Lolium rigidum* L.) has already shown resistance to glyphosate in Australia (Pratley et al., 1999). Thus, herbicide resistance of weeds is a major threat to sustainable crop production. Consequently, alternatives to conventional synthetic herbicide application have become a focus of much research in Australia and worldwide. The potential use of crop allelopathy as part of a weed control program is one option gaining attention of the researchers (Kathiresan, 2005).

Rice (1984) defined allelopathy as the direct or indirect (harmful or beneficial) effect of a plant, and microbes, on another plant through the release of compounds into the environment. Allelochemicals have usually been considered to be secondary metabolites or waste products of the main metabolic pathways in plants (Swain, 1977) and released via several mechanisms (Seigler, 1996; Singh et al., 2003; Weston and Duke, 2003) including leaching (by dew and rain), residue decomposition (Putnam and DeFrank, 1983; Purvis et al., 1985) and exudation from living plants (Rice, 1984; Blum, 2011; Thorpe et al., 2011). Furthermore, the production and the release of biologically active compounds differ between species and between cultivars (Jeffery et al., 2003; Bennett et al., 2006; Keurentjes et al., 2006; Abdel-Farid et al., 2007), although relatively few have strong allelopathic properties (Bhowmik and Inderjit, 2003; Khanh et al., 2005; Xuan et al., 2005). The potential role of crop allelopathy in weed control has been the focus of much research and has been extensively reviewed (e.g., Einhellig and Leather, 1988; Purvis, 1990; Wu et al., 1999). Results from allelopathic assessment of canola cultivars against weeds *in vitro* and under field condition showed that canola allelopathy is genetically controlled (Asaduzzaman et al., 2014a,b). Canola allelopathy also seems to be independent from the competitive traits in the above ground morphology growth and phenology of the crop (Asaduzzaman et al., 2014c,d). However, there are no reports that holistically analyze the canola allelochemicals complex.

Plant secondary metabolites are generally present in plant tissue but few are exuded into the environment (Weston and Duke, 2003; Badri and Vivanco, 2009). To establish the involvement of any root exudates in crop plant allelopathy, it is important to demonstrate their phytotoxic effect by direct release to the growth medium (Inderjit, 1996). The exudation of allelochemicals by plant roots is an active metabolic process (Overland, 1966) and seems to be universal in the plant kingdom (Martin, 1957; Fay and Duke, 1977; Abdul-Rahman and Habib, 1989; Einhellig and Souza, 1992). Brassicaceae plants possess several groups of secondary metabolites including phenylpropanoids (hydroxycinnamates), flavonoids, as well as Brassicaceae-specific metabolites such as glucosinolates. The characterisation of these phytochemicals between strong and weak allelopathic cultivars is very important, as it will help to understand the chemical basis of canola allelopathy. Appropriate advanced tools, such as metabolomics, can be used for identifying and characterizing the potential metabolites responsible for the allelopathic defenses recently demonstrated in canola (Asaduzzaman et al., 2014a,b).

Metabolomics is an approach that allows a biochemical analysis of the total metabolite complement of a given plant tissue (Rinu et al., 2005; Kim et al., 2011). It is being used as an important procedure for identifying compounds involved in allelopathic interactions (D’Abrosca et al., 2013). Through mass spectral (MS) analysis of metabolomes in plant organs and principal component analysis (PCA), relative variability between organs can be explored. In addition, due to complex interactions, the field assessment of crop allelopathy is challenging (Inderjit and del Moral, 1997; Olofsdotter et al., 1999; Inderjit and Weston, 2000; Bertin et al., 2003; Bais et al., 2006) and difficult to separate from competition (Olofsdotter et al., 1999). Hence, laboratory screening of crop cultivars, coupled with advanced multivariate statistical analysis of metabolomes, offers new insights into the subterranean biology of plant allelopathy (Rinu et al., 2005).

The present research aimed to determine the metabolite composition of different organs (namely shoot, root) and root exudates of canola by using time-of-flight (TOF–MS) analysis technique and to establish a platform for understanding canola allelopathy.

## MATERIALS AND METHODS

###  

#### Plant materials

Six canola (*Brassica napus*, rapeseed, oilseed rape) genotypes were selected for this study namely: Av-opal, Pak85388-502, Av-garnet, Barossa, Cb-argyle and Atr-409. Previous field and *in vitro* screening results showed that Av-opal and Pak85388-502 were strongly allelopathic against annual ryegrass *in vitro,* and against the background weed populations (over 2 years: 2012 and 2013) under field conditions, whereas, Atr-409 and Barossa were weakly allelopathic genotypes (Asaduzzaman et al., 2014a,b). Two other genotypes were chosen based on a previous canola competitiveness field study conducted by Lemerle et al. (2014): Av-garnet was reported to be strongly competitive and Cb-argyle weakly competitive on weed species and associated total weed biomass. Seeds of these canola genotypes were obtained from the National *Brassica* Germplasm Improvement Program, located at NSW Department of Primary Industries, Wagga Wagga, NSW, Australia. Agar (technical grade) was purchased from Sigma Aldrich (St. Louis, MO, USA).

#### Sterilization and germination

Canola seeds were surface-sterilized by soaking in 2% sodium hypochlorite (NaOCl) for 5 min, then rinsed six times in sterilized distilled water. The seeds were transferred to a petri dish with one sheet of Whatman No. 1 filter paper, moistened with 5 ml sterilized distilled water, and sealed with parafilm. The surface-sterilized seeds of *Brassica* and ryegrass were kept in a 12-h light/12-h dark, 20/15°C controlled environment for 36 h and 48 h respectively.

#### General bioassay and growing conditions

The equal-compartment-agar-method (ECAM), described previously by Wu et al. (2000a) was chosen for bioassay. The method was developed based on the plant box method and relay seedling technique and separates competition and allelopathy phenomena between two simultaneously growing species. In this method, each species was placed into separate regions in the same container, where each species received equal space for its root system development. Briefly, glass beakers (600 mL, 12 cm depth, 8 cm diameter) containing 30 mL of 0.3% agar-medium (no nutrients, 1.3 cm depth) were autoclaved. The previous bioassay of 70 canola genotypes showed that 30 seedlings/beaker allelopathically gave greatest inhibition of the root length of annual ryegrass (Asaduzzaman et al., 2014a). Hence for each genotype, 30 uniform seedlings per beaker were chosen and aseptically transplanted from the germination dish onto one half of the agar surface, with the embryo up. The beaker tops were sealed with parafilm to prevent contamination and evaporation from the agar surface, and the beakers were placed in a controlled growth incubator with a daily 12-h light/12-h dark, 20/15°C cycle. Canola plants were grown for 6 days, 15 pre-germinated uniform seeds of annual ryegrass were aseptically sown on the other half of the agar surface at a distance of 4 cm from the canola seedlings. A piece of pre-autoclaved white paperboard was inserted across the center and down the middle of the beaker with the lower edge of the paperboard kept 1 cm above the agar surface. The beaker was divided into two equal compartments to minimize competition for space and light between the canola and ryegrass seedlings. The roots of canola freely entered the ryegrass compartment so that any allelochemicals produced and released by the canola seedlings can diffuse throughout the entire agar medium to influence ryegrass root growth. After ryegrass sowing, the beakers were again wrapped with parafilm and placed back in the growth chamber for 7 days. The receiver species, annual ryegrass, was also grown alone as a control. After 7 days, each annual ryegrass and canola seedling was carefully removed from the agar to avoid root breakage, and the root and the shoot lengths of 10 randomly selected plants within each beaker of both species were measured.

#### Experimental design and statistical analysis

A randomized complete block design was used for the experiment described. For each genotype 30 replicates were used in three different experimental units (beakers). The inhibited root and shoot length of annual ryegrass (mm) was converted as percentage of control. To determine the percentage change a percent ration was calculated between the mean root/shoot length of all (*n* = 30) ryegrass seedlings and the root/shoot length of every singly seedling. Further, to evaluate the equivalence of shoot and root inhibition of ryegrass with root length of canola, Pearson correlation co-efficient values were calculated. A linear regression analysis (*y* = mx + c) was also performed between root length (mm) of canola (independent) and of annual ryegrass (dependent) to know their mutual relationship. All data were subjected to analysis of variance using Genstat v13 (VSN International, Hemel Hempstead, UK) and the treatment means compared using the least significance difference (LSD) at a 5% level of probability. Plots of residual versus fitted values were examined for all traits to ensure the normality and homogenecity.

### BIOCHEMICAL ANALYSIS BY METABOLOMICS APPROACH

#### Preparation of shoot and root extracts

Canola seedlings of each genotype were grown alone at a density of 30 seedlings/beaker for 13 days, as described in the above laboratory bioassay (see General bioassay and growing conditions). The roots and the shoots were cut from the canola seedlings and were immediately stored at -80°C in a sealed container. The frozen tissue was then freeze-dried for 24 h (Alpha 2–4 LD plus; John Morris). To extract metabolites, the freeze-dried tissue was then crushed to a fine powder using liquid nitrogen-chilled mortar and pestle. Sixty mg of the root and the shoot tissue of each canola genotype were placed separately into a 2 mL tube chilled in liquid nitrogen. The tube was filled with 400 μL 100% methanol solution containing internal standards ^13^C_6_-sorbitol (0.5 mg/mL); ^13^C_5_^15^N-valine (0.5 mg/mL); penta-fluorobenzoic acid (0.25 mg/mL) and 2-aminoanthracene (0.25 mg/mL; Roessner and Dias, 2013). The tubes were vortexed for 30 s and centrifuged for 15 min at 13000 rpm at 4°C. The supernatant was transferred to a new pre-labeled 2 mL tube. An amount of 400 μL MQ water was added to the remaining pellet and vortexed, centrifuged and the supernatant was combined with the previous methanol containing supernatant. Three aliquots of each tissue containing 650 μL were prepared and stored at -80°C until analysis.

#### Collection of root exudates

Canola seedlings were carefully uprooted from their nutrient-free agar medium and the roots were rinsed twice with 5 mL portions of distilled water to remove any adhering agar and root exudates. The washings were pooled with the agar medium (30 mL). The agar medium was stirred carefully and extracted three times using 5 mL of 80% methanol. The extracted samples were vortexed and centrifuged and filtered through a 0.22 μm syringe filter into 2mL labeled tubes. Three aliquots of 650 μL of each genotype were prepared and stored at -80°C before analysis.

#### Metabolites profiling by LC-QTOF-MS

To assess the metabolite composition differences among the organs and root exudates of canola genotypes, non-targeted and targeted metabolite profiling of extracted material was conducted. The compounds of canola shoots, roots extracts, and root exudates were separated on an Agilent 6520 LC-QTOF-MS system (Santa Clara, CA, USA, Agilent Mass Hunter Qualitative Analysis Build 6.0), with a dual sprayer ESI source, and attached to an Agilent 1200 series HPLC system (Santa Clara, CA, USA) consisting of a vacuum degasser, binary pump, with a thermo stated auto-sampler, column compartment, and diode array detector. The MS was operated in the negative mode using the following conditions: nebuliser pressure 45 psi, gas flow-rate 10 L/min, gas temperature 300°C, capillary voltage 3500V, fragmentor 150 and skimmer 65V. The instrument was operated in the extended dynamic range mode with data collected in mass-to-charge ratio (m/z), range 70–1700 amu.

#### Chromatography

An Agilent Zorbax Eclipse XDB-C18, 2.1 × 100 mm, 1.8 μm (Agilent) column was used with a flow rate of 400 μL/min maintained at ambient temperature (35 ± 1°C), resulting in operating pressures below 600 bar with a 12 min run time. A gradient LC-QTOF-MS method (**Table 1**) was used with mobile phases comprised of (A) 0.1% formic acid in de-ionized water and (B) 0.1% formic acid in acetonitrile. The sample run was conducted first for the 5 min by using linear gradient from 5% solvent (B) to 30% solvent (B), followed by a 5 min linear gradient to 30% solvent (B) to 100% solvent (B), then a 2 min hold at 100% solvent (B) and a 5 min re-equilibration at 5% solvent (B). Total time = 17 min. Three replications were run for each category of samples of each genotype.

**Table 1:** Gradient of LC Method for 6520-QTOF.

Time (min)	A%	B%
0.00	95.0	5.0
5.00	70.0	30.0
10.00	0.0	100.0
12.00	0.0	100.0
12.10	95.0	5.0
17.00.	95.0	5.0

#### Mass spectrum data processing

Relative qualitative analyses of the metabolites in the six canola genotypes were performed using Mass Hunter data analysis software (Agilent Technologies, USA). The extracted molecular features of each detected compound were matched with two different data bases (METLIN-AM-PCDL and HMDB-KEGG), plus the mass of the reference compounds from commercial standards. The individual compounds were also determined through assessing the outcomes of score (>70), hit count (total number of hits in the database) and mass differences (<5.0).

### CHEMOASSAYS USING REFERENCE COMPOUNDS

#### Preparation of the different concentrations

Stock solutions (10000 μM) of sinapyl alcohol, *p*-hydroxybenzoic acid and 3,5,6,7, 8-pentahydroxy flavones were prepared separately. A mixture of these three compounds (10000 μM) was also made by using 1:1:1 ratio. The stock solutions of individual compounds and of their mixture were diluted to concentrations of 5000, 100, and 50 μM in HPLC-grade methanol.

#### Annual ryegrass bioassay with reference compounds

The modified chemical bioassay described by Seal et al. (2004b) was used to evaluate the phytotoxic effects of three reference compounds on annual ryegrass. One milliliter of each of the above concentrations (50, 100, 5000 and 10000 μM) was added to 600 ml beakers lined with Whatman #1 filter paper (Micro science, grade: MS 2 85 mm, size: 85 mm, Quality: 100) at the base. For the control, 1 ml of pure methanol was added. After the methanol had completely evaporated using the method described by Seal et al. (2004b), 5 ml of sterile double distilled water was added. Ten annual ryegrass seeds were sown directly into the water and the beaker was covered with parafilm. Three replicates of each treatment were arranged in a randomized complete block design in a growth chamber described in “General bioassay and growing conditions.” 7 days later the annual ryegrass seedlings were removed from the system and both their root and shoot lengths were measured to the nearest 0.5 mm.

#### Statistical analysis

All dose-response curves were subjected to two-way ANOVA using Genstat v13 (VSN International, Hemel Hempstead, UK). Annual ryegrass root length (mm) was converted as percentage of control as described in “Experimental design and statistical analysis.” The treatment means were compared using the LSD at a 5% level of probability. Plots of residual versus fitted values were examined for all traits to ensure that the assumptions of analysis of variance were met.

## RESULTS

### LABORATORY BIOASSAY

**FIGURE 1:**
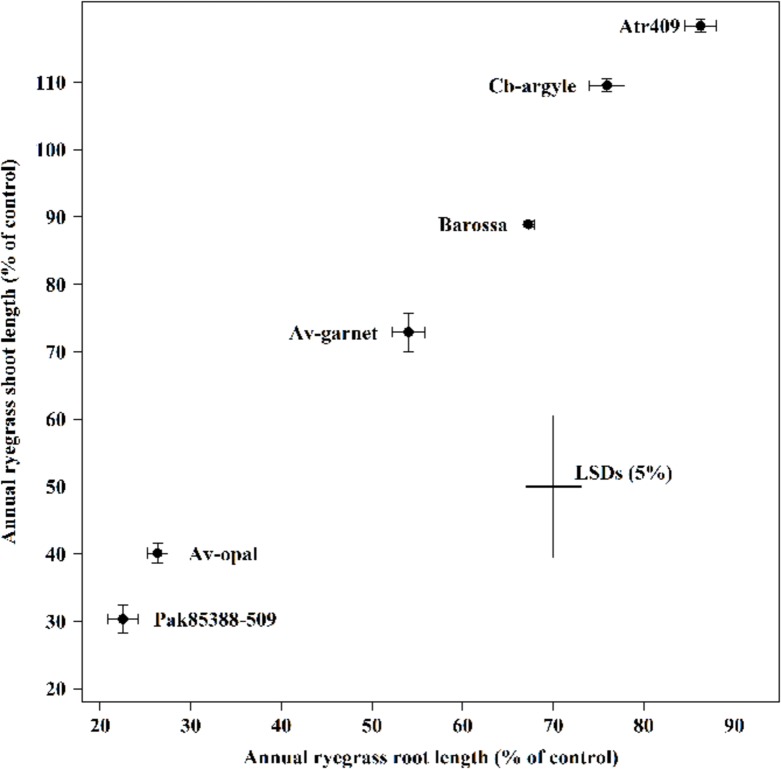
**Laboratory bioassays (ECAM) of canola (*Brassica napus*) seedling allelopathy against annual ryegrass (*Lolium rigidum*) seedlings.** Data shown are the means (*n* = 30; ±SE) of the root and the shoot length (% of control) of annual ryegrass seedlings. Data were pooled of three experimental units (beakers). The cross indicates significant difference between the genotypes within two variables (root and shoot; *P* < 0.05, ANOVA with *post hoc* Fisher-LSD-test). The Pearson correlation coefficient (*r*) is 0.99 (*P <* 0.001).

Genotypes differed significantly (*P* < 0.001) in their ability to suppress the root and the shoot growth of annual ryegrass (**Figure 1**). Genotypes Atr-409, Cb-argyl and Barossa showed less inhibitory effects on annual ryegrass while Av-opal, Pak85388-502 and Av-garnet were more inhibitive. In all collections, root growth (14–76%) of annual ryegrass was inhibited more than shoot growth (0–15%). The most suppressive genotype Pak85388-502 resulted in 76% root growth control of annual ryegrass followed by genotype Av-opal (74%) and Av-garnet (46%). The weakest genotype cv. Atr-409 inhibited the root length of annual ryegrass by only about 14%.

**FIGURE 2:**
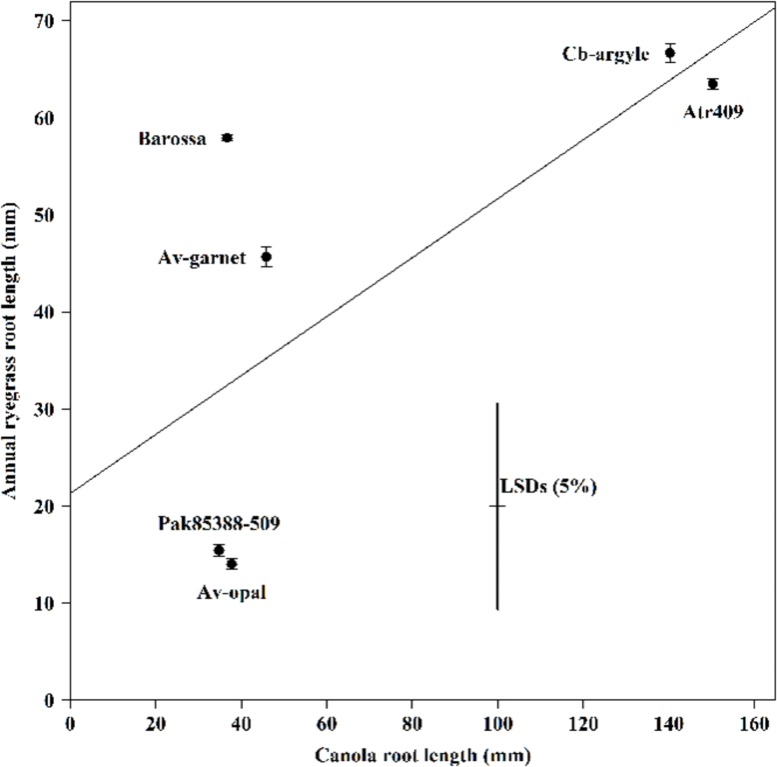
**The root length of canola (*Brassica napus*) and annual ryegrass (*Lolium rigidum*) seedlings when grown together in the ECAM bioassay.** Data shown are means (*n* = 30;±SE) of the root length (mm) of annual ryegrass seedlings and of the root length (mm) of canola seedlings. Data were pooled of three experimental units (beakers). The cross indicates significant differences between the genotypes within one variable (*P* < 0.05, ANOVA with *post hoc* Fisher-LSD-test). The Pearson correlation coefficient (*r*) is -071 (*P* > 0.05) and were determined between the two variables across all genotypes and the regression equation is *y* = 0.303x + 21.33, with *R*^2^ = 0.50, *P* > 0.05).

The average root length of canola seedlings differed significantly (*P* < 0.001) between genotypes (**Figure 2**). Genotypes Av-opal and Pak85388-502 produced the longest root; in contrast Cb-argyle and Atr-409 produced the shortest roots. The regression analysis (*r* = -0.71; *y* = 0.303x + 21.33) showed that annual ryegrass root growth (mm) was not decreased (*P* > 0.05) with increased canola root growth (mm).

### METABOLITE PROFILING

The different metabolite patterns were observed by simple visual inspection of the MS traces of the three different organs. A total of 2806 mass signals were recorded in three different sample types. The number of metabolites in the root and the shoot extracts varied between genotypes. Metabolites were highly enriched in root extracts followed by shoot extracts and root exudates (**Table 2**). Over 1807 compounds were found in roots, with Av-opal, Pak85388-502, Barossa and Atr-409 assigned 1586, 1532, 1471 and 1525 compounds respectively.

**Table 2:** Total numbers of metabolites identified in root and shoot extracts and root exudates of six canola genotypes.

	Number of metabolites		
Genotype	Root extracts	Shoot extracts	Root exudates
Av-opal	1586	1494	908
Pak85388-502	1532	1496	951
Av-garnet	1436	1498	774
Barossa	1471	1402	920
Cb-argyle	1525	1524	888
Atr-409	1479	1479	957
Mean	1505	1480	899
LSD, *P* < 0.001	29	33	71

### IDENTIFICATION OF PHYTOCHEMICALS IN CANOLA GENOTYPES

Fourteen secondary metabolites, including two internal signaling molecules, namely jasmonic acid and methyl-jasmonate, were detected across the samples of the six canola genotypes (**Table 3**). Only eight metabolites were identified in the root exudates.

**Table 3:** Phytochemicals identified in shoot and root extracts and root exudates of six canola genotypes using LC-QTOF-MS in negative mode and matched with data from two data bases.

SL	Name	Formula	RT (min)	Mass	Score	m/z	Shoot extracts*	Root extracts*	Root exudates*
1	Malonic acid	C_3_ H_4_ O_4_	0.696	104.011	73.64	104.01095	3, 4, 6	4, 6	4, 6
2	Isocitric Acid	C_6_ H_8_ O_7_	0.931	192.0259	97.45	192.0210	1, 2, 3, 4, 5, 6	1, 2, 3, 4, 5, 6	1, 2, 3, 4, 5, 6
3	2-hydroxy-3,4-dimethoxybenzoic acid	C_9_ H_8_ O_4_	4.857	180.043	76	180.04225	1, 2, 3, 4, 5, 6	1, 2, 3, 4, 5, 6	–
4	Sinapyl alcohol	C_11_ H_14_ O_4_	4.987	210.087	94.06	210.08920	1, 2, 3, 4, 5, 6	1, 2, 3, 4, 5, 6	1, 2
5	Rutin	C_27_ H_3__0_ O_16_	5.002	610.1559	78.19	610.15338	1, 2, 3, 4, 5, 6	1, 2, 3, 4, 5, 6	–
6	*p*-hydroxybenzoic acid	C_7_ H_6_ O_3_	5.348	138.0303	78.37	138.03169	–	1, 2, 3, 4, 5, 6	1, 2
7	Vanillic acid	C_8_ H_8_ O_4_	5.59	168.0414	81.29	168.04225	1, 2, 3, 4, 5, 6	1, 2, 3, 4, 5, 6	–
8	trans-3-hydroxycinnamic acid	C_9_ H_8_ O_3_	6.356	164.0458	73.8	164.0473	1, 2, 3, 4, 5, 6	–	–
9	Dimethoxy-4-hydroxycinnamic acid	C_11_ H_12_ O_5_	6.631	224.0693	98.34	224.06847	–	1, 2, 3, 4, 5, 6	1, 2, 4, 6
10	2-phenylethyl glucosinolates	C_9_H_9_NS	6.832	163.24	90.03	163.04556	2, 4,5	2, 4, 5	–
11	Quercitin	C_15_ H_10_ O_7_	7.159	302.046	69.87	302.04265	1, 2, 3, 4, 5, 6	1, 2, 3, 4, 5, 6	3
12	3,5,6,7,8 pentahydroxy flavone	C_15_H_10_ O_7_	7.50	302.205	70.05	302.04265	–	1,2	1, 2
13	Jasmonic acid	C_12_ H_18_ O_3_	8.224	210.1224	81.95	210.12559	–	1, 2, 3, 5	–
14	Methyl jasmonate	C_13_ H_20_ O_3_	9.541	224.1386	72.05	224.14124	–	1, 2	1, 2

*Number indicates whether the compound is found in the tissue of the six genotypes: 1, Av-opal; 2, Pak85388-502; 3, Av-garnet; 4, Barossa; 5, Cb-argyle; 6, Atr-409; “–,” not present.

The three interested metabolites were only found in the root exudates of highly allelopathic genotypes (Av-opal, Pak85388-502, and possibly Av-garnet). Five metabolites (or some mixture of these) were the most likely candidates for an allelopathic effect; sinapyl alcohol, *p*-hydroxybenzoic acid, quercitin, 3,5,6,7,8- pentahydroxy flavones, and methyl-jasmonate. Of these five, quercitin was formed only in the exudates of Av-garnet, and sinapyl alcohol was found only in the exudates of Av-opal and Pak85388-502.

### CHEMOASSAYS USING REFERENCE COMPOUNDS

**FIGURE 3:**
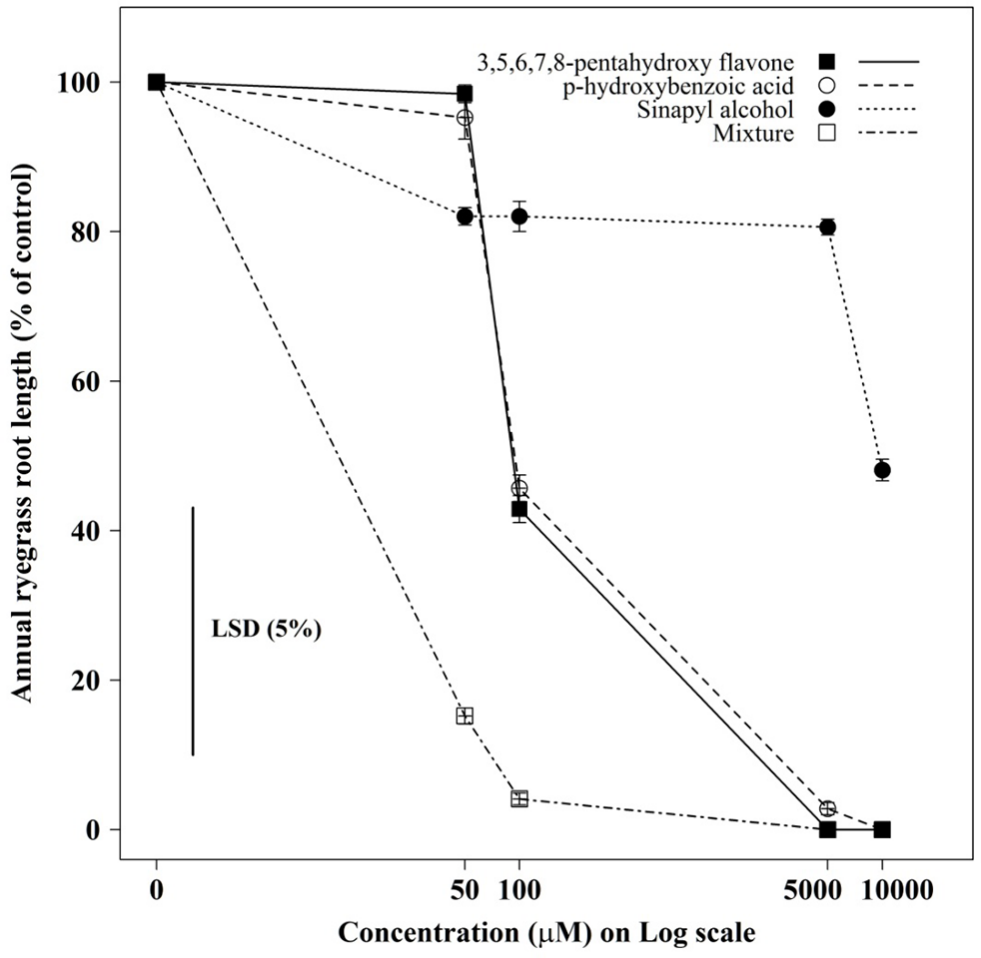
**The individual, and combined, effect of sinapyl alcohol, *p*-hydroxybenzoic acid, and 3,5,6,7,8-pentahydroxy flavone on the root growth of annual ryegrass seedlings.** Data shown are the means (*n* = 30; ±SE) of the root length of annual ryegrass seedlings. Data were pooled of three experimental units (beakers). The cross indicates significant difference between the genotypes × concentration interaction within one variables (*P* < 0.05, ANOVA with *post hoc* Fisher-LSD-test).

**FIGURE 4:**
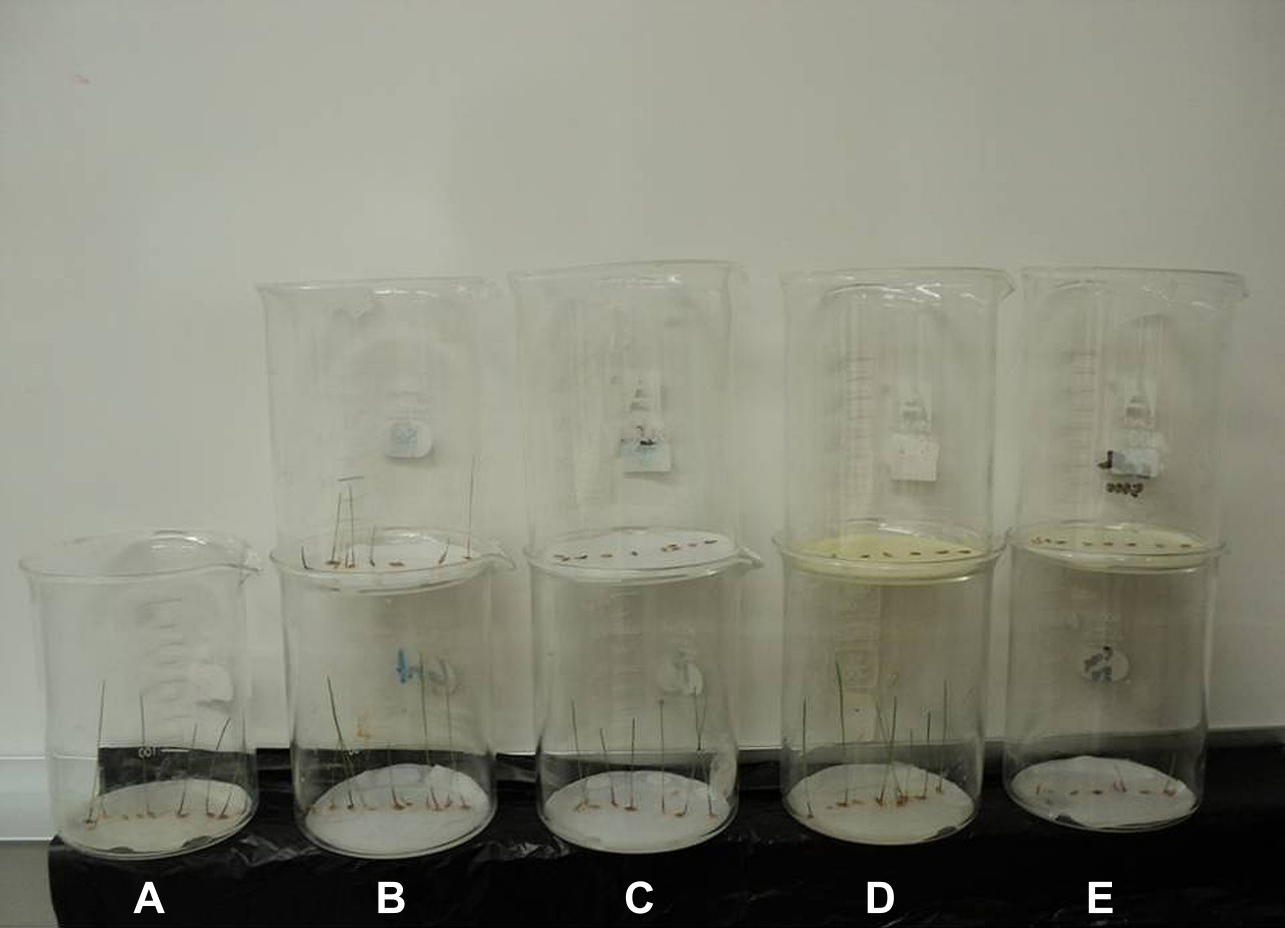
**Comparison of the seedlings growth of annual ryegrass affected by **(A)** control, **(B)** sinapyl alcohol, **(C)***p*-hydroxybenzoic acid, **(D)** 3,5,6,7,8-pentahydroxy flavone and **(E)** their mixture.** The beakers in the bottom were treated with low concentration (50 μM), while beakers on the top were treated with high concentration (10000 μM).

The root growth of annual ryegrass seedlings differed significantly (*P* < 0.001) between compounds and their concentrations (**Figures 3** and **4**). Among the compounds 3,5,6,7,8- pentahydroxy flavones showed greater toxicity, while sinapyl alcohol was less toxic in all tested concentrations. When all tested compounds were considered together in mixture, the root growth of ryegrass was inhibited more compared to the individual effect of each compound, even in medium concentrations. Under the mixture of three compounds at, medium-to-high concentrations (100μM–10000μM) the germination ability of most of the ryegrass seeds was restricted.

## DISCUSSION

Different inhibition activities against ryegrass seedlings were observed among the tested canola genotypes. This is in accordance with previous observations in rice (Seal et al., 2004a), wheat (Wu et al., 2000b), and rapeseed (Uremis et al., 2009), leading to the general conclusion that allelopathy is genetically controlled. The most allelopathic genotypes in this study were Av-opal and Pak85388-502, then competitive genotype Av-garnet. This suggests that root exudation from Av-opal and Pak85388-502 might also have played a significant role for its allelopathic activity in the bioassay. These two genotypes were previously characterized as highly allelopathic *in vitro* testing (Asaduzzaman et al., 2014a) and were also highly weed suppressive in the field (Asaduzzaman et al., 2014b).

The negative relationship between the root length of canola and annual ryegrass suggests that long roots of canola seedlings might produce more allelochemicals than short roots. Hence, despite vigorous shoot growth, Barossa and Av-garnet showed less root-exuded allelopathic activity, whereas the short vegetative growth but longer root growth of Av-opal still inhibited the root growth of annual ryegrass to a greater extent. Such findings also infer that the inhibition effects on the receiver plant were due to chemical interactions between the roots and that such chemicals were exuded into the agar by the canola roots. It seems possible that the allelopathy potential of any particular genotype depends upon firstly, the chemical composition of the root exudates, and secondly, the amount of chemical exuded which may be a function of root system length or surface area particular at later growth satge.

The biochemical analysis of canola organs and root exudates showed differences between genotypes in the production of their total metabolomes. It is to be expected that different canola genotypes will produce varying types and amounts of phytotoxic compounds since this has been shown to occur in various other crop species (Guenzi and McCalla, 1966; Fay and Duke, 1977; Wu et al., 2001; Jeffery et al., 2003; Fang et al., 2012; Farag et al., 2012). Gardiner et al. (1999) reported that the roots of rapeseed (*B. napus* L) contained more compounds than did the shoot. The root also contributed more to the total chemical pool for allelopathic activity (Gardiner et al., 1999). Similarly, in this study, the number of metabolites was generally higher in the root than in the shoot and in root exudates. Allelopathic research findings have also revealed that the allelochemical concentrations were higher in the roots than in the shoots of wheat (Wu et al., 2001). It is not clear whether the higher amounts of these allelochemicals in the roots result from their direct synthesis *in situ,* from their translocation from the shoots to the roots, or both. The presence of chemicals in the root exudates does not infer that they play any role in the observed phytotoxicity. However, it suggests that roots and shoots contain many compounds but only some are released as root exudates, depending upon particular conditions in the rhizosphere (Badri and Vivanco, 2009).

In previous *Brassica* allelopathy research, glucosinolates and their derivatives were proposed as potential allelochemicals of the crop’s residue (Gardiner et al., 1999). These compounds were detected only in the root and the shoot extracts of three genotypes in this study. Possibilities for their non-detection in root exudates include: they remained locked inside the vacuole of fresh tissue of living plant; or they could not be detected due to their complex volatile nature. Glucosinolates were not detected in the root exudates from living tissue of any genotypes showing high allelopathy in our study. Therefore it seems unlikely that they are responsible for allelopathy. This conclusion is most striking when comparing the consistent results from the three replications of the tested genotypes, including Av-opal and Pak85388-502. Both are highly allelopathic but Av-opal is low in glucosinolates in the seed while Pak85388-502 is high in glucosinolates in the seed (Asaduzzaman et al., 2014b). Glucosinolates and their breakdown products are significant in the phytotoxic effects observed for canola stubble and stubble leachates after harvest (Boydston and Hang, 1995; Brown and Morra, 1996; Al-Khatib et al., 1997). It may be that senescence (aging) and fallen leaves may make a contribution to weed suppression during the life cycle of the crop but this has not been specially recorded. The cut and green manure rapeseed suppressed weeds (Boydston and Hang, 1995) but this may be due to physical smothering rather than chemical effects.

Several potential allelopathic compounds were found in the root and the shoot tissue in this study but were not detected in root exudates. This suggests that the expression of the allelopathic effect not only depends on particular compounds being synthesized but also on the ability of the genotypes to actively exude these into their rhizosphere. For instance, Barossa and Atr-409, the two weakly allelopathic genotypes, contained potential phytotoxic metabolites in the roots and the shoots but their inhibitory effect on annual ryegrass was weak. Dicarboxylic malonic acid was found only in the root exudates of these two weakly allelopathic genotypes and this compound may act as a buffering agent to reduce the threshold levels of other potential allelochemicals in the rhizosphere. Similar results have been also reported in rice (Seal et al., 2004b), where the amounts of dicarboxylic acids was high in root exudates of non-allelopathic rice cultivars.

Sinapyl alcohol, *p*-hydroxybenzoic acid and 3,5,6,7,8-pentahydroxy flavone were isolated from root exudates of the two strongly allelopathic canola genotypes, suggesting that they were at least partly responsible for the observed allelopathic activity. The detection of two signal molecules (jasmonic acid and methyl-jasmonate) in the allelopathic genotypes also supports the proposition that they are also involved in canola allelopathy. Jasmonic acid and methyl-jasmonate act as secondary messengers in signal transduction events in the cell and have inhibitory effects on many plant physiological processes (Sembdner and Parthier, 1993). Abdel-Farid et al. (2007) reported that the accumulation of these signal molecules is connected with demand or synthesis of the secondary metabolites sinapyl alcohol and *p*-hydroxy benzoic acid in *Brassica rapa*. Furthermore, 3,5,6,7,8- pentahydroxyflavone was also detected previously in root exudates of another member of the Brassicaceae, *Brassica alba* (Ponce et al., 2004). *p*-hydroxybenzoic acid has been reported as a potential allelochemical in other crops including, *Glycine max* (Barkosky and Einhellig, 1993), *Camelina alyssum* (Grummer and Beyer, 1960), and several members of the genus *Althaea* (Gude and Bieganowski, 1990). Some of the reduction in root and coleoptile growth of wheat seedlings caused by wild oat (*Avena fatua*) root exudates is attributed to this compound (Perez and Ormeno-Nunez, 1991).

It has been postulated that allelopathic effects are most likely due to the combination and interaction of a complex mixture of compounds (Rizvi and Rizvi, 1992; An et al., 2001). The chemobioassay results of the present study revealed that, the allelopathic activity of canola cultivars resulted from the synergistic effects of sinapyl alcohol, *p*-hydroxybenzoic acid and 3,5,6,7,8-pentahydroxy flavones. It is possible that multiple compounds present at low concentrations can have pronounced allelopathic effects through their joint action, though evidence for this elusive. Joint allelopathic interactions between compounds have also been reported in several tested species including rice (Chou et al., 1991; Seal et al., 2004b) and vulpia (An et al., 2001).

The phytotoxicity observed among the tested canola genotypes indicates that allelopathy plays a role in inhibiting the annual ryegrass weed species. Field experiments (Asaduzzaman et al., 2014b) support this conclusion. The comprehensive chemical analysis reported here revealed that sinapyl alcohol, *p*-hydroxybenzoic acid and 3,5,6,7,8-pentahydroxy flavones in most suppressive genotypes (cv. Av-opal and Pak85388-502) are likely allelopathic agents via root exudates in canola.

## Conflict of Interest Statement

The authors declare that the research was conducted in the absence of any commercial or financial relationships that could be construed as a potential conflict of interest.
